# Sp1/3 and NF-1 mediate basal transcription of the human *P2X*_1 _gene in megakaryoblastic MEG-01 cells

**DOI:** 10.1186/1471-2199-7-10

**Published:** 2006-03-10

**Authors:** Jiangqin Zhao, Steven J Ennion

**Affiliations:** 1 Department of Cell Physiology and Pharmacology, University of Leicester, PO Box 138, Leicester, UK

## Abstract

**Background:**

P2X_1 _receptors play an important role in platelet function as they can induce shape change, granule centralization and are also involved in thrombus formation. As platelets have no nuclei, the level of P2X_1 _expression depends on transcriptional regulation in megakaryocytes, the platelet precursor cell. Since nothing is known about the molecular mechanisms regulating megakaryocytic P2X_1 _expression, this study aimed to identify and functionally characterize the *P2X*_1 _core promoter utilized in the human megakaryoblastic cell line MEG-01.

**Results:**

In order to identify cis-acting elements involved in the transcriptional regulation of *P2X*_1 _expression, the ability of 4.7 kb *P2X*_1 _upstream sequence to drive luciferase reporter gene expression was tested. Low promoter activity was detected in proliferating MEG-01 cells. This activity increased 20-fold after phorbol-12-myristate-13-acetate (PMA) induced differentiation. A transcription start site was detected 365 bp upstream of the start codon by primer extension. Deletion analysis of reporter constructs indicated a core promoter located within the region -68 to +149 bp that contained two Sp1 sites (named Sp1a and Sp1b) and an NF-1 site. Individual mutations of Sp1b or NF-1 binding sites severely reduced promoter activity whereas triple mutation of Sp1a, Sp1b and NF-1 sites completely abolished promoter activity in both untreated and PMA treated cells. Sp1/3 and NF-1 proteins were shown to bind their respective sites by EMSA and interaction of Sp1/3, NF-1 and TFIIB with the endogenous *P2X*_1 _core promoter in MEG-01 cells was demonstrated by chromatin immunoprecipitation. Alignment of *P2X*_1 _genes from human, chimp, rat, mouse and dog revealed consensus Sp1a, Sp1b and NF-1 binding sites in equivalent positions thereby demonstrating evolutionary conservation of these functionally important sites.

**Conclusion:**

This study has identified and characterized the *P2X*_1 _promoter utilized in MEG-01 cells and shown that binding of Sp1/3 and NF-1 to elements in the direct vicinity of the transcription start site is essential for basal transcription. Targeting the function of these transcription factors in megakaryocytes may therefore provide a basis for the future therapeutic manipulation of platelet P2X_1 _function.

## Background

P2X receptors comprise a family of membrane cation channels gated by extracellular ATP. Seven subtypes (P2X_1–7_), each encoded for by a separate gene, are present in mammalian species (reviewed by [1]) and these combine to form either homo or hetero trimeric channels [2,3] exhibiting different expression profiles and functional properties. P2X_1_, the first subtype to be isolated, was cloned over a decade ago [4] and the physiological roles of this channel in the regulation of arterial tone [5] and in the neuronal control of smooth muscle contraction [6,7] are well established. The presence of P2X_1 _receptors in platelets was discovered shortly after its cloning [8,9], however, it has not been until more recent times that the physiological importance of P2X_1 _in platelet function has started to fully emerge (for review see [10]). Activation of platelet P2X_1 _receptors by extracellular ATP results in a rapid Ca^2+ ^influx which activates cytoskeletal events to induce a transient but substantial shape change and granule centralization [11,12], both important early events in platelet activation. P2X_1 _receptors can also synergize with P2Y, collagen, adrenaline and thrombopoietin receptor mediated responses [10] to enhance platelet function. Furthermore, over expression of P2X_1 _in the platelets of transgenic mice results in a prothrombotic phenotype [13] whereas an absence of the receptor in P2X_1 _knockout mice leads to a reduced risk of thrombosis [14] suggesting that the level of P2X_1 _receptor expression in platelets is of physiological and clinical significance.

Although the physiological roles and consequences of altered expression levels of platelet P2X_1 _receptors are becoming increasingly apparent, the mechanisms that regulate P2X_1 _expression in platelets are poorly understood. Since platelets are cell fragments devoid of any nuclei the complement of channels and receptors present on their surface must ultimately rely on the regulation of gene transcription in the megakaryocyte, the platelet precursor cell. bHLH factors and E proteins are thought to be involved in smooth muscle specific *P2X*_1 _transcription [15]. Nothing however is known about transcriptional regulation of *P2X*_1 _in megakaryoblastic cells. In order to initiate studies on the transcriptional mechanisms involved in determining P2X_1 _expression levels in platelets, we have identified and characterized the *P2X*_1 _promoter in the megakaryoblastic cell line MEG-01 [16]. We show that Sp1 and NF-1 regulatory elements located in the direct vicinity of the transcriptional start site are essential for basal transcription of *P2X*_1 _in MEG-01 cells.

## Results

### Cloning and analysis of the *P2X_1 _*gene 5' upstream sequence

The human genomic BAC clone RP11-167N20 was found to contain all 12 exons of the human *P2X*_1 _gene and 64 kb 5' upstream sequence. The 4.772 kb upstream of the ATG start codon was cloned into the pGL-3 Basic luciferase reporter plasmid to generate the plasmid p-4407/+365 and the sequence analyzed by the Proscan computer program [17]. A potential core promoter was predicted at -151 to +99 bp with a promoter score of 70.32.

### The *P2X*_1 _transcription start site is located 365 bp upstream of the start codon

**Figure 1:**
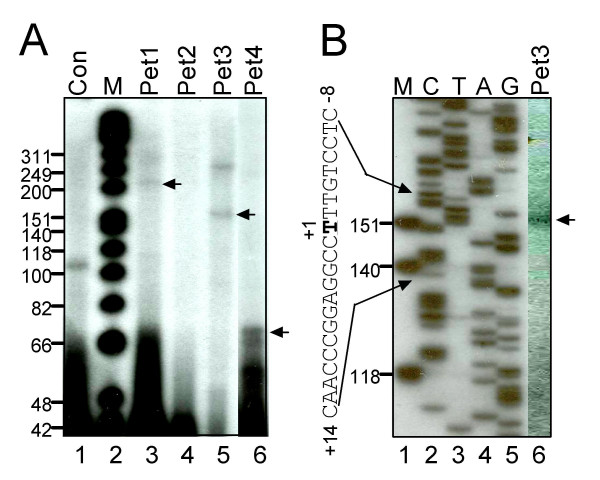
**The *P2X*_1 _transcription start site is located 365 bp upstream of the start codon**. A) ^32^P-labelled primers Pet1, Pet2, Pet3 and Pet4 and Con (control primer pTXRHR-100) were annealed to poly(A) RNA from PMA-treated MEG-01 cells and extended with reverse transcriptase. Extension products for Pet1, Pet3 and Pet4 that equate to the same transcription start site are indicated with arrows. (M), molecular mass marker. B) Pet3 extension reaction (lane 6) run in parallel with a sequencing ladder. The location of primers is depicted in Figure 3.

**Figure 2:**
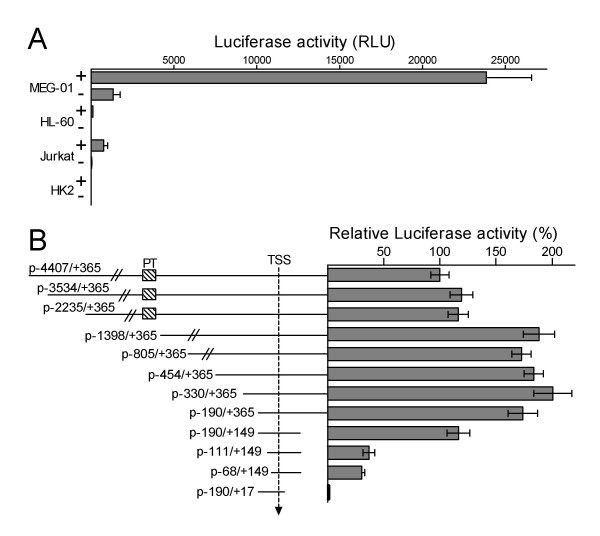
**Activity of *P2X*_1 _promoter constructs in transient transfection assays**. A) Promoter activity of p-4407/+365 was determined both with (+) and without (-) the prior treatment of cells with 10 nM PMA. Data are means of absolute firefly luciferase activity of three independent transfections. B) Luciferase activity of *P2X*_1 _deletion constructs in PMA treated MEG-01 cells. TSS, transcription start site. PT depicts a polypyrimidine tract located from -1801 to -1405 bp. Values are presented as means of at least three experiments and normalized against the construct p-4407/+365. Names of plasmid constructs correspond to the 5' and 3' ends of the insert relative to the transcription start site e.g. construct p-4407/+365 extends from 4407 bp 5' to 365 bp 3' of the transcription start site.

**Figure 3:**
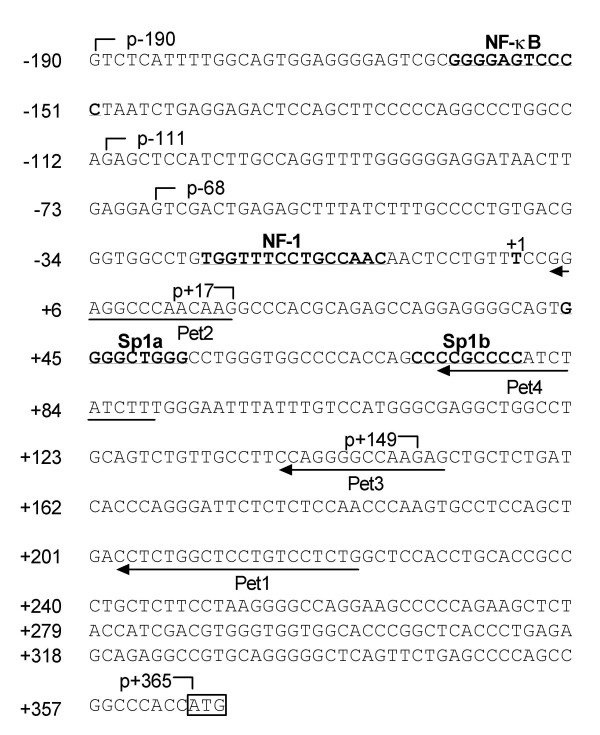
**Sequence of the *P2X*_1 _proximal promoter region**. Potential transcription factor binding sites are underlined and in bold. Oligonucleotide sequences used for primer extension analysis (Pet1-4) are indicated by horizontal arrows. End points of plasmid deletion constructs in this region are indicated by "p" and number above the sequence. Sequence numbering is relative to the transcription start site indicated as +1. The translation initiation ATG is boxed. The EMBL/Genbank accession number of this sequence is AJ971536.

To map the location of the *P2X*_1 _transcription initiation site in MEG-01 cells, four anti-sense oligonucleotides Pet1, Pet2, Pet3 and Pet4 (Figure 3) were used for primer extension. As positive control we utilized a previously published primer sequence pTXRHR-100 which has been used to map the transcription start site of the thromboxane receptor gene in MEG-01 cells [18]. Similar to previous studies, this control primer yielded a single extension product of ~110 bp (Figure 1A lane 1). A single extension product of ~210 bp was generated by Pet1 (Figure 1A lane 3), two distinct extension products of ~150 bp and ~249 bp, were generated by Pet3 (Figure 1A lane 5), a doublet band of ~66 bp was generated by Pet4 (Figure 1A lane 6) and no extension products were observed from Pet2 (Figure 1A lane 4). Extension products of 66 bp (Pet4), 150 bp (Pet3) and 210 bp (Pet1) correspond to a transcription start site located ~365 bp upstream of the ATG start codon. The Pet2 sequence is very close to this site (Figure 3) and so would not be expected to produce an extension product. An additional extension product of 249 bp produced by the Pet3 primer (Figure 1A, lane 5) indicated the possibility of an additional transcription start site located ~465 bp upstream of start codon. However, no bands corresponding to such a start site were observed with the Pet1, Pet2 or Pet4 primers. Furthermore, subsequent analysis of promoter activity in constructs where this potential site had been removed (p-111/+149 compared to p-68/+149 (Figure 2B)) did not indicate the presence of a functional transcription start site in this region. We therefore attributed the 249 bp band produced by the Pet3 primer as a non-specific extension product. To precisely map the start site, sequencing reactions primed with the same Pet primer used for primer extension were run in parallel to extension reactions (Figure 1B). The 150 bp Pet3 extension product (Figure 1B lane 6) corresponds to a transcription start site located at a "T" residue 365 bp upstream of the ATG start codon. This site is 78 bp downstream of the start site previously described for *P2X*_1 _in smooth muscle [15] but matches closely to the EST clone BX644575 which was isolated from human bone marrow, a tissue rich in megakaryocytes.

### PMA induced differentiation results in a 20-fold increase in *P2X*_1 _reporter gene activity

In initial studies, we tested the p-4407/+365 construct for promoter activity in three cell lines previously reported to show endogenous P2X_1 _expression, MEG-01 [9,19], HL-60 [20] and Jurkat [21]. The human kidney proximal tubular epithelial cell line HK2 was used as negative control. Cells were assayed both with and without prior treatment with PMA, a phorbol ester previously reported to increase P2X_1 _mediated currents in MEG-01 [9,19] and HL-60 [20] cells. Low promoter activity was detected in untreated MEG-01, HL-60 and Jurkat cells but not in HK2 cells (Figure 2A). Treatment of MEG-01 cells with 10 nM PMA caused cells to differentiate to a non-proliferating adherent phenotype and resulted in a ~20 fold increase in promoter activity of p-4407/+365. This PMA-induced increase was also apparent in Jurkat and HL-60 cells but not in HK2 cells (Figure 2A). Comparisons of promoter activity between different constructs in untreated MEG-01 cells proved problematic since promoter activities were not sufficiently above background to allow accurate quantification. PMA-differentiated MEG-01 cells were therefore utilized for subsequent analysis since these cells represent a well characterized model of megakaryocyte maturation [22] and the higher *P2X*_1 _promoter activity aided quantification of differences between constructs.

### Deletion analysis of luciferase reporter gene constructs

To define the mechanisms controlling transcriptional regulation of *P2X*_1 _in MEG-01 cells, a series of deletion constructs were generated. Gradual deletion of 5' sequence from -4407 to -2235 resulted in no significant change in luciferase activity (Figure 2B) suggesting that no important regulatory elements reside in this ~2 kb region. Removal of sequence between -2235 and -1398 in construct p-1398/+365 however resulted in a 88 ± 14% increase (p < 0.01) in promoter activity indicating the presence of a repressive element. Sequence analysis of this region revealed a 396 bp polypyrimidine tract located -1801 to -1405 bp which contains 15 copies of the sequence motif 5'-TCCCTCCCTCCC-3'. Interestingly, this same sequence motif is present in the mouse c-Ki-ras promoter where it plays a role in transcriptional regulation [23]. Within this polypyrimidine tract region are clustered multiple potential Sp1, Ets-2 and MAZ transcription factor binding sites. The increase in promoter activity was maintained throughout deletions from 1398 to 190 in the constructs p-805/365, p-454/+365, p-330/+365, and p-190/+365 (p < 0.01 for each construct) (Figure 2B). The difference in promoter activity between constructs p-1398/+365 through to p-190/+365 was not significant. Removal of 3' sequence from +365 to +149 bp (p-190/+149) decreased promoter activity back to the level of the p-4407/+365 construct. Deletion of 5' sequence from -190 to -111 bp (p-111/+149) resulted in a 63 ± 5% decrease (p < 0.01) in promoter activity. Further deletion of 43 bp 5' sequence in construct p-68/+149 produced no significant change in activity from p-111/+149. Removal of 3' sequence however from +149 to +17 bp in construct p-190/+17 completely abolished promoter activity.

Thus, to summarize the deletion analysis performed on the *P2X*_1 _upstream region, promoter activity was stable or increased in 5' deletions from -4407 through to position -190. Deletions past -190 up to position -68 significantly reduced promoter activity whereas removal of 132 bp from +149 to +17 bp abolished promoter function demonstrating that this region contains regulatory elements essential for transcription.

### *In vitro *binding of Sp1/3 and NF-1 to the *P2X*_1 _promoter

**Figure 4:**
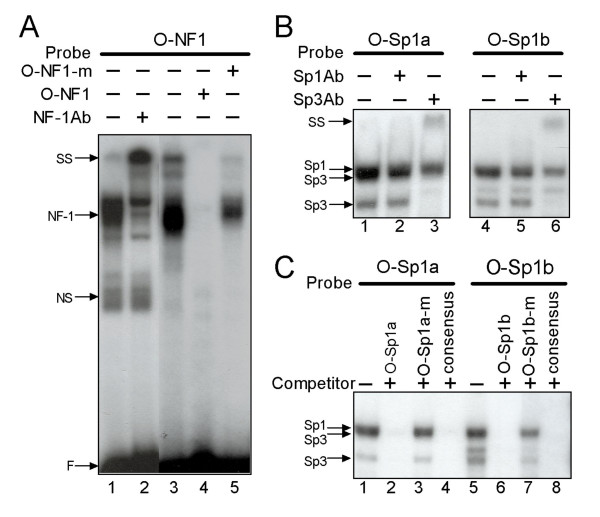
**EMSA assays on NF-1 and Sp1/Sp3 binding sites**. A) Competition experiments were performed in the presence of excess unlabelled O-NF1 (lane 4) and unlabelled mutated O-NF1-mut (lane 5). Complexes supershifted by pre-incubation with an NF-1 antibody (NF-1Ab, lane 2) are marked "SS". "NS" indicates non specific binding complexes, the level of which varied between individual nuclear extractions (lanes 1 and 3). "F" indicates free probe. B) Supershift assays using Sp1 (lanes 2 and 5) and Sp3 (lanes 3 and 6) specific antibodies. C) Competition experiments in the absence (lanes 1 and 5) or presence of excess of unlabelled competitors: Lanes 2 and 6, an excess of unlabelled O-Sp1a or O-Sp1b probe respectively. Lanes 3 and 7, an excess of unlabelled mutated oligonucleotide O-Sp1a-mut and O-Sp1b-mut respectively. Lanes 4 and 8, an excess of a consensus Sp1 oligonucleotide (Promega, E323A).

Results from the above deletion analysis suggested that the region -190 to +149 bp plays an important role in the transcriptional regulation of the *P2X*_1 _gene (Figure 3). Analysis of this region using the programmes TESS [24] and PROSCAN [17] did not reveal putative TATA or CCAAT boxes, but detected several potential transcription factor binding sites including NF-κB, YY-1, GATA-1, NF-1, and multiple Sp1 sites. To begin to understand which specific transcription factors are involved in regulating *P2X_1 _*gene transcription we initially performed EMSAs on nuclear extracts from PMA-treated MEG-01 cells using oligonucleotides corresponding to a potential NF-1 site and two Sp1 sites (named Sp1a and Sp1b) located in the immediate vicinity of the transcription start site. The radiolabelled probe O-NF1 containing a potential NF-1 binding site produced a major retarded band (Figure 4A lanes 1 and 3), the formation of which was prevented by an excess of unlabelled O-NF1 itself (Figure 4A lane 4) but not by the mutated O-NF1-mut probe (Figure 4A lane 5). In the presence of an anti-NF-1 antibody, the DNA-protein complexes were either supershifted or remained in the well (Figure 4A lane 2). Similar results for NF-1 supershift complexes have also been reported in previous studies [25,26], we therefore concluded that NF-1 can bind to the *P2X*_1 _proximal promoter.

The probes O-Sp1a and O-Sp1b corresponding to the Sp1a and Sp1b binding sites respectively, gave rise to three major DNA-protein complexes, the larger two of which appear as a doublet band (Figure 4B lanes 1 and 4). To identify specific transcription factor binding, supershift assays with Sp1 and Sp3 antibodies were performed. The Sp3 antibody produced a supershift ("ss" Figure 4B lanes 3 and 6). Sp1 antibodies caused a decrease in intensity of the doublet band but produced no visible supershifted products (Figure 4B lanes 2 and 5). To further clarify the molecular basis of Sp1/Sp3 interactions with the oligonucleotide probes, competition experiments were performed (Figure 4C). Addition of unlabelled O-Sp1a or O-Sp1b prevented binding of Sp1/Sp3 to labelled probe (Figure 4C lanes 2 and 6). Similarly, addition of an unlabelled commercial consensus Sp1 oligonucleotide (Figure 4C lanes 4 and 8) also prevented Sp1/Sp3 binding to O-Sp1a and O-Sp1b. In contrast O-Sp1a-mut and O-Sp1b-mut did not prevent protein binding (Figure 4C lanes 3 and 7) demonstrating that a 2 bp mutation (Table 1) disrupted Sp1/3 binding.

An oligonucleotide probe corresponding to the potential NF-κB binding site (O-NFκB) did not produce super-shifted products after addition of an NF-κB antibody (data not shown). In summary EMSAs demonstrated that Sp1, Sp3 and NF-1 transcription factors can bind regulatory elements in the P2X_1 _proximal promoter implying that these factors are involved in the regulation of P2X_1 _gene transcription.

### Sp1 and NF-1 sites are essential for basal transcription of the *P2X*_1 _gene

**Figure 5:**
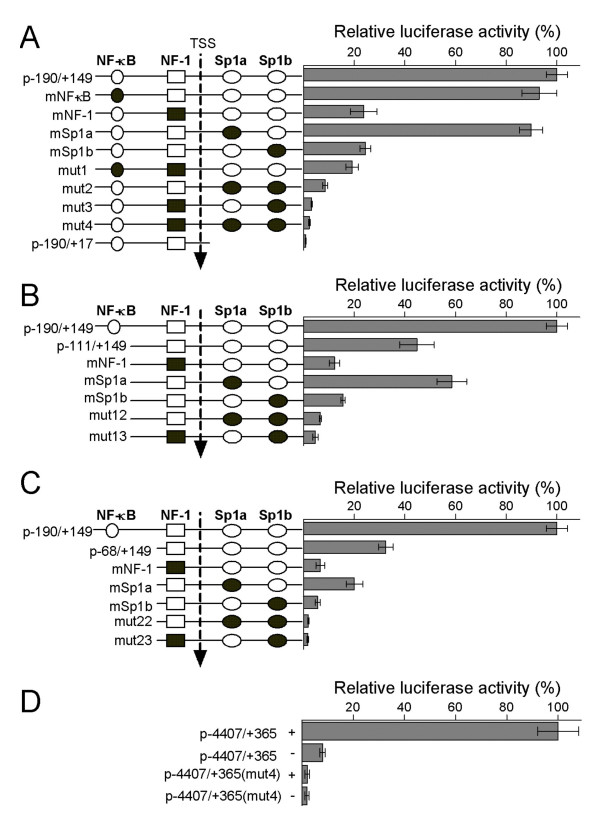
**Mutation analysis of transcription factor binding sites**. A series of luciferase reporter constructs harbouring site-directed mutations in the NF-κB, NF-1 and Sp1 binding sites of the *P2X*_1 _proximal promoter region were assayed for promoter activity in PMA-treated MEG-01 cells (sequences of mutations are shown in Table 2). Shaded symbols represent mutated binding sites. Values are presented as means ± S.E of at least three independent experiments and are normalized against the wild type p-190/+149 construct for panels A to C and the wild type construct p-4407/+365 in panel D. A) Effects of mutations in the construct p-190/+149. B) Effect of mutation after removal of the potential NF-κB binding site. C) Effect of mutation after removal of the transcription start site that is utilized by smooth muscle cells [15]. Dotted vertical arrow (TSS) represents the transcription start site determined in this study. D) The Sp1a, Sp1b and NF-1 sites were mutated in the original full length 4.7 kb reporter construct p-4407/+365 to generate the triple mutated construct p-4407/+365(mut4). Promoter activities were determined in MEG-01 cells either treated (+) or untreated (-) with PMA.

We next conducted site-directed mutagenesis to disrupt the Sp1/3, NF-1 and NF-κB binding sites in selected *P2X*_1 _promoter constructs. Promoter activity of mutated constructs was determined in PMA treated MEG-01 cells and expressed relative to that of the wild type p-190/+149 construct (Figure 5). Single mutation of the NF-κB (construct mNF-κB) and Sp1a (construct mSp1a) sites produced no significant change in promoter activity (Figure 5A). Single mutation of the NF-1 site (construct mNF-1) or Sp1b site (construct mSp1b) however resulted in 76 ± 5% (p < 0.01) and 76 ± 2% (P < 0.01) reductions respectively. A double mutation ablating both NF-1 and NF-κB sites simultaneously (construct mut1) reduced promoter activity by 80 ± 2% (p < 0.01), whereas double mutation of Sp1a and Sp1b sites (construct mut2) reduced activity by 92% ± 1 (p < 0.01). Simultaneous mutation of NF-1 and Sp1b sites (construct mut3), the NF-1 site together with Sp1a and Sp1b sites (construct mut4), or deletion of the fragment from +18 to +149 bp which removes both Sp1a and Sp1b sites (construct p-190/+17) effectively abolished promoter activity (Figure 5A).

In order to confirm the apparent redundancy of the potential NF-κB site, we generated a further set of mutations in the plasmid p-111/+149, a construct where the potential NF-κB site has been removed (Figure 5B). Whilst removal of 79 bp from -190 to -111 resulted in a ~50% decrease in promoter activity, the overall pattern of promoter activity resulting from Sp1 and NF-1 binding site mutations was very similar to that observed in the p-190/+149 mutations (Figure 5A).

A *P2X*_1 _transcription start site 78 bp upstream from site identified in this current study has been reported in human smooth muscle cells [15]. If this same site is utilized in MEG-01 cells then its removal in deletion constructs would be expected to abolish promoter activity. To test this hypothesis and to further delineate a functional core promoter, the construct p-68/+149 was generated. p-68/+149 displayed a promoter activity 32 ± 3% that of the p-190/+149 construct. Single and double site-directed mutations of Sp1 and NF-1 binding sites in this p-68/+149 construct again showed a similar pattern of promoter activity to that observed in the p-190/+149 construct (Figure 5C).

From the above mutation and previous deletion analysis, we defined the region spanning -68 to +149 as the *P2X*_1 _core promoter composing of a single NF-1 binding site and two Sp1-binding sites. To prove the importance of these sites in controlling basal transcription, the Sp1a, Sp1b and NF-1 sites were mutated in the original 4.7 kb reporter construct p-4407/+365 to generate the construct p-4407/+365(mut4). Promoter activity from this triple mutated construct was abolished in both untreated and PMA-treated MEG-01 cells (Figure 5D) confirming the essential roles of Sp1 and NF-1 in *P2X*_1 _basal transcription.

### Direct binding of Sp1/3 and NF-1 to the endogenous *P2X*_1 _core promoter

**Figure 6:**
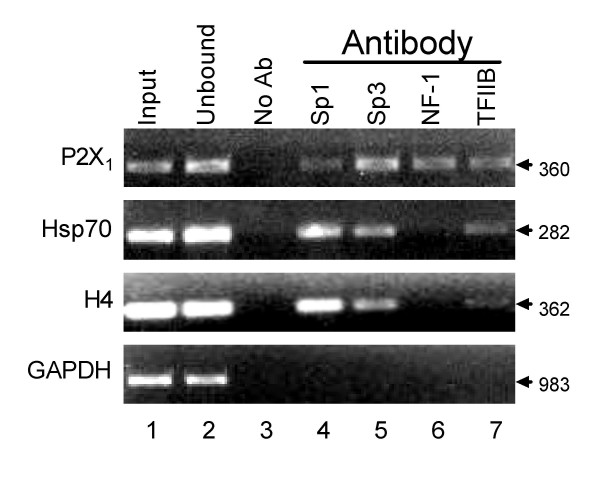
**Transcription factors bind the endogenous *P2X*_1 _promoter**. Formaldehyde cross-linked chromatin was prepared from PMA-treated MEG-01 cells and immunoprecipitated with antibodies to Sp1 (lane 4), Sp3 (lane 5), NF-1 (lane 6), TFIIB (lane 7), or in the absence of antibody (lane 3). PCR was performed with specific primers for the *P2X*_1_, *H4 *and *Hsp70 *promoter and for the *GAPDH *coding region as a negative control. A sample representative of the total input chromatin (input DNA lane 1) was included in the PCR analysis. Lane 2 shows the supernatant of the 'unbound' sample. PCR products were between 282 and 983 bp in length. PCR cycle numbers were 31 for the *P2X*_1_, *H4 *and *Hsp70 *promoter and 36 for the *GAPDH *coding region. Primer sequences are given in Table 3.

Having identified Sp1, Sp3 and NF-1 sites essential for *P2X*_1 _reporter gene function, we next utilized ChIP to determine whether the corresponding transcription factors are physically recruited to the endogenous *P2X*_1 _core promoter *in vivo *(Figure 6). Primers for the *Hsp70 *and *histone H4 *promoters were used as positive controls as Sp1 and Sp3 have previously been detected at these promoters by ChIP assay [27,28]. As a negative control, parallel experiments using primers for the coding region of the *GAPDH *gene were performed. Antibodies for Sp1, Sp3, NF-1 and TFIIB in ChIP assays using chromatin isolated from PMA-treated MEG-01 cells resulted in enrichment of the DNA genomic segment harboring the region -197/+166 of the *P2X*_1 _gene thus confirming the *in vivo *recruitment of these factors to the core promoter.

## Discussion

Regulation of *P2X*_1 _transcription in megakaryocytic cells is of physiological and clinical importance as ultimately this will influence the level of P2X_1 _receptor present on platelets, excessive activation of which can lead to thrombosis, cardiovascular disease and stroke. As a first step towards understanding the transcriptional mechanisms regulating *P2X*_1 _expression in megakaryocytic cells, we have analyzed the *P2X*_1 _promoter in MEG-01 cells. We identified a transcription start site 365 bp upstream of the start codon and defined a core promoter located within the region -68 to +149 bp. Two Sp1 sites and an NF-1 site in the core promoter were shown to be critical for basal transcription.

The *P2X*_1 _core promoter was found not to possess "TATA" or "CCAAT" elements but contained Sp1 and NF-1 sites critical for basal transcription. Mammalian promoters lacking a TATA box often contain Sp1 elements and rely on Sp1 to recruit TATA-binding protein and guide RNA polymerase II to the initiator site [29-31]. Sp1 and Sp3 are members of a large family of transcription factors that regulate transcription through interactions with GC-boxes. They are both zinc finger proteins that bind the same DNA recognition sequences with similar affinity [32]. Despite ubiquitous expression, tissue-specific gene activation by Sp1 and Sp3 may be achieved by a variety of mechanisms including variability in the concentration of Sp1 and Sp3 [32], interactions of Sp1 with tissue specific transcription factors [33] and tissue specific post-translational modification of Sp1 and Sp3 [32]. Sp1/3 can regulate gene transcription through a variety of mechanisms, functioning as either a basal promoter element or an upstream activator, depending on promoter [34,35]. Two Sp1/3 sites were identified in the *P2X*_1 _promoter and mutation of the Sp1b (+71 to +79) site most markedly reduced promoter activity. Single mutation of the Sp1a (+44 to +52) site did not significantly reduce transcription. However, simultaneous mutation of both Sp1a and Sp1b together entirely abolished transcriptional activity. Thus, the Sp1b site can completely compensate for the loss of the Sp1a site but Sp1a can only partially compensate for the loss of Sp1b suggesting that whilst both sites may be utilized in basal transcription, the Sp1b site plays a more prominent role.

An NF-1 site in the *P2X*_1 _core promoter was also found to be important for transcription. NF-1 proteins are recruited to promoters in a cell type specific manner and can associate with different regulators to either activate or repress transcription depending on the promoter and cellular context [36,37]. Site-directed mutagenesis of the NF-1 site in the *P2X*_1 _core promoter significantly reduced transcriptional activity by ~80% (Figure 5). Simultaneous mutation of both NF-1 and Sp1b sites completely abolished promoter activity suggesting that NF-1 and Sp1/3 may act synergistically to recruit the initiation complex to the *P2X*_1 _core promoter.

The human *P2X*_1 _promoter identified in this study shows characteristics typical of those normally associated with housekeeping genes: lack of classical TATA or CCAAT boxes, an increased GC content and critical Sp1 and NF-1 elements [38]. This would appear ideally suited for P2X_1 _expression in megakaryocytes since constant synthesis is required for platelet production. Constitutive expression of *P2X*_1 _however does not occur in all hematic cell types and their progenitors as neutrophils, monocytes and lymphocytes show no significant expression of P2X_1 _[39,40]. This suggests that tight repression of the P2X_1 _promoter occurs and that release from this repression may be an important feature of *P2X*_1 _transcriptional regulation. Deletion analysis performed in this study identified a repressive element in an upstream region containing a 396 bp polypyrimidine tract located -1801 to -1405. Removal of this tract in deletion constructs p-1398/+365 through to p-190/+365 resulted in a ~90–100% increase in promoter activity (Figure 2B). Polypyrimidine tracts can harbor multiple binding sites for Sp1 and Sp3 [41,42] and play important roles in the regulation of gene transcription [43,44]. A possible mechanism for the repressive effect of the *P2X*_1 _polypyrimidine tract could be that this region facilitates a local sequestration of Sp1/3 factors hindering their binding to the Sp1a and Sp1b sites further downstream in the core promoter.

**Figure 7:**
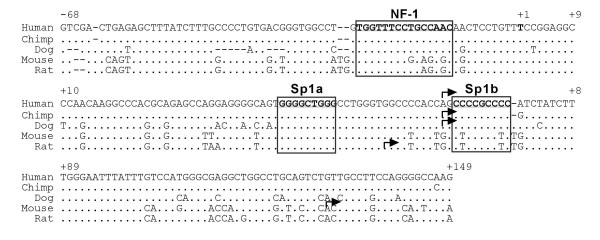
**Cross species conservation of the *P2X*_1 _core promoter**. P2X_1 _upstream sequence from chimp (chromosome 19 position 4051445–4051944), dog (chromosome 9 position 40295005–40295504), rat (chromosome 10 position 59889637–59890136) and mouse (chromosome 11 position 72611373–72611872) was obtained by BLAST searching of genomic databases via the ENSEMBL genome browser . The 500 bp upstream of the ATG start codon of each gene was aligned using CLUSTAL. Alignment equivalent to -68/+149 of the human *P2X*_1 _core promoter is shown. Sequence identity to the human P2X_1 _sequence over this region was 99.1%, 83.4%, 76.8%, and 75.9% for chimp, dog, mouse and rat respectively. "." indicates nucleotides identical to the human P2X_1 _sequence. Dashes indicate positions where gaps have been inserted in order to maintain the alignment. The transcription start site of the human P2X_1 _gene determined in this study is indicated as +1. Potential transcription start sites predicted by PROSCAN [17] are indicated as arrows. In each species, potential NF-1, Sp1a and Sp1b binding sites (indicated by boxes) are present in equivalent positions to the human P2X_1 _core promoter.

Important transcriptional regulatory elements often show conservation between species [45]. We therefore investigated whether the NF-1, Sp1a and Sp1b binding sites identified in the human promoter are also present in the upstream sequence of *P2X*_1 _genes from other species. Using BLAST searches of genomic sequence data we identified *P2X*_1 _genes from mouse, chimp, dog and rat. Similar to human, the upstream regions of these genes were also GC-rich and contained no "TATA" or "CCAAT" elements. Sequence alignment revealed NF-1, Sp1a and Sp1b sites in identical positions across all species examined (Figure 7). These results demonstrate a conserved core promoter structure in mammalian P2X_1 _genes and suggest that the involvement of Sp1/3 and NF-1 in the regulation of *P2X*_1 _transcription has been conserved during evolution.

PMA-induced differentiation of MEG-01 cells is widely used as a model of megakaryocytic maturation and is known to increase the expression of megakaryocytic lineage specific antigens, such as integrin α_IIb_β_3 _[22,46]. PMA activates signaling cascades via activation of PKC and other C1 domain containing receptors resulting in a wide range of cellular effects [47]. PMA can enhance P2X_1 _mediated responses in MEG-01 [19], THP1 [39], and HL-60 [20] cells and is also thought to have more acute effects by increasing phosphorylation of a putative P2X_1 _accessory protein [48]. In this study, we found that *P2X*_1 _promoter activity increased ~20-fold in PMA-differentiated MEG-01 cells compared to untreated cells. Several studies have shown Sp1 DNA-binding activity and expression level to increase upon treatment of cells with PMA [49-53]. Similarly, PMA induction has been shown to increase the formation of NF-1 DNA complexes [54,55]. We therefore speculate that PMA enhances P2X_1 _ion channel function through the actions of Sp1 and NF-1 at the *P2X*_1 _core promoter resulting in an increase in gene transcription and the synthesis of new P2X_1 _protein. The signaling pathways that link PMA stimulation with *P2X*_1 _gene activation and whether these result in alterations in the levels of Sp1 and NF-1 or in protein modifications such as phosphorylation altering the affinity of factors for their respective binding sites remain to be determined.

## Conclusion

The *in vivo* control of *P2X_1_* transcription in megakaryocytes is undoubtedly a complex combinatorial process involving the integration of a large number of protein-protein and protein-DNA interactions that mediate cell type specific expression during megakaryocyte differentiation and the ability respond to regulatory inputs. This study provides the first steps towards unraveling the complexities of this control by providing the fundamental information of *P2X_1_* core promoter location and composition in a megakaryocytic cell line. We demonstrate that Sp1 and NF-1 are critical for *P2X_1_* core promoter function in both proliferating and PMA-differentiated MEG-01 cells. The targeting of signaling pathways that influence the function of these transcription factors in megakaryocytes may therefore provide the basis for the future therapeutic manipulation of platelet P2X_1 _function.

## Methods

### Reporter constructs

The human genomic BAC clone (RP11-167N20) containing the human *P2X*_1 _gene was obtained from the Sanger Institute. 4.772 kb upstream of the start codon was amplified by PCR using *Pfu *polymerase (Promega, Madison, WI) and cloned into pGL3-Basic (Promega). A series of 5' deletion constructs were created either by restriction enzyme digestion with Mlu I/Hind III or via a PCR based approach to introduce Mlu I/Hind III sites via primers.

### Cell culture

MEG-01, HL-60 and Jurkat cells were purchased from the German National Resource Centre for Biological Material (DSMZ) and maintained at 37°C in 5% CO_2 _in RPMI 1640 medium supplemented with 10% heat-inactivated fetal bovine serum. HK2 cells were maintained in Dulbecco's modified Eagle's medium-Ham's F12 mix supplemented with 10% fetal bovine serum and 2 mM L-glutamine.

### Site-directed mutagenesis

Point mutations were introduced into potential Sp1 and/or NF-1 binding sites by a two step cloning strategy. A 339 bp fragment (spanning from -190 to +149 bp) was cut from the *P2X*_1 _pGL3-Basic plasmid, cloned into the Mlu I/Hind III sites of pCR-Script™ and used as template for mutagenesis using the QuikChange™ system (Stratagene, La, Jolla CA). Mutated fragments were then re-cloned into the Mlu I/Hind III sites of the corresponding pGL3-Basic vectors. Double (mut1, mut2 and mut3) and triple-site (mut4) mutation constructs were generated by consecutive rounds of mutagenesis. The triple mutation construct p-4407(mut4) was generated by replacing a Sal I/Hind III fragment with the equivalent mutated Sal I/Hind III fragment from the plasmid mut4.

### Mapping the *P2X*_1 _transcription start site

Poly(A) RNA was isolated from PMA (10 nM) treated MEG-01 cells using RNAWIZ™ total RNA and Poly(A) Purist™ mRNA purification kits (Ambion, Austin, TX). Extension of [γ-^32^P]ATP labeled primers (Figure 1) was performed using the Promega Primer Extension System (Promega, E3030). Size of extension products was determined by resolution on denaturing 8% polyacrylamide gels. Locations of the primers used are depicted in Figure 3.

### Transient transfection and luciferase assays

For transient transfections 5 × 10^4 ^cells were plated in 96-well plates and incubated with 80 ng of *P2X*_1 _reporter plasmid, 20 ng of pRL-SV40 and 0.3 μl of transfection reagent (GeneJuice™, Novagen Madison, WI). The Renilla luciferase plasmid pRL-SV40 (Promega) was used as internal control for transfection efficiency. Following transfection, cells were cultured in the absence or presence of 10 nM PMA. The Dual-Glo™ Luciferase Assay (Promega) was used to determine firefly and renilla luciferase activity 48 hours after transfection in 96-well plates. Firefly luciferase activity was normalized to Renilla activity in each transfection. All plasmids were purified using Qiagen columns (Qiagen, Valencia CA) and at least two preparations per plasmid were tested. Transfections were done in triplicate in each experiment and at least 3 experiments were performed for each construct. Data are presented as means of experiments ± Standard error. Differences between means of control and experimental constructs were assessed by Dunnett's test. Differences between experimental constructs within a dataset were assessed using Tukey's multiple comparison test.

### Electrophoretic mobility shift assays

Nuclear proteins were isolated from PMA-treated (10 nM, 48 hours) MEG-01 cells essentially as described by Hurst [56]. EMSAs were performed using the Promega EMSA kit on at least three different preparations of nuclear extract for each experiment. Bands that could not be competed by cold unlabelled oligonucleotide, consensus oligonucleotide or the non-specific competitor Poly dI-dC were classed as non-specific binding complexes. Nuclear protein (5.0 μg) was incubated with or without a 100-fold molar excess of unlabelled competitor DNA in 4% glycerol, 1 mM MgCl_2_, 0.5 mM dithiothreitol, 0.5 mM EDTA, 50 mM NaCl, 10 mM Tris-HCl, pH 7.5 and 2.0 μg poly (dI-dC) on ice for 15 minutes, followed by addition of the radiolabelled probe. For supershift assays, antibodies (Santa Cruz Biotechnology Inc, Santa Cruz, CA) were added to the reaction mixture 25 minutes prior to the probe. DNA-protein complexes were resolved by electrophoresis on 6% polyacrylamide gels. Commercial double-stranded oligonucleotides used were as follows: Sp1, 5'-ATTCGATCGGGGCGGGGCGAGC-3', (Promega); NF-1, 5'-TTTTGGATTGAAGCCAATATGATAA-3' (Santa Cruz). Other oligonucleotide sequences are shown in Table 1.

### Chromatin Immunoprecipitation

Formaldehyde cross-linking and chromatin immunoprecipitation were performed as described by Okada and co-workers [57]. After PMA treatment for 48 hours, 2 × 10^6 ^cells were treated with formaldehyde for 10 minutes followed by the addition of 0.125 M glycine. Cells were washed twice in PBS and re-suspended in lysis buffer (1% SDS, 10 mM EDTA, 50 mM HEPES, 10 μl/ml Protease inhibitor Cocktail (Sigma) adjusted to pH 7.9 with KOH). After sonication to an average fragment size of 500 bp, protein-DNA cross-linked products were enriched by immunoprecipitation. Samples without antibody were included as negative controls. After reversal of cross links and DNA purification, the extent of enrichment was monitored by PCR using primers to *P2X*_1_, *H4*, and *Hsp70 *promoter fragments and to the coding region of *GAPDH *as negative control. The input sample was processed with the rest of the samples from the point at which the cross links were reversed.

## Abbreviations

EMSA, electrophoretic mobility shift assay; PMA, phorbol-12-myristate-13-acetate; S.E, standard error; ChIP, chromatin immunoprecipitation.

## Authors' contributions

JZ carried out the experimentation and planned the study. SJE cloned the full length *P2X_1_* reporter construct, planned the study and wrote the manuscript.

Both authors read and approved the final manuscript

## Figures and Tables

**Table 1:** Oligonucleotides used for EMSA. Primer sequences are 5' to 3'. Bold lowercase bases represent mutations with respect to the wild type sequence. Underlined sequences represent consensus binding sites for respective transcription factor.

**Name**	**Position**	**Sequence**
O-NFκB	-168/-144	GAGTCGCGGGGAGTCCCCTAATCTG
O-NFκB-mut		GAGTCGCGG**cc**AGTCCCCTAATCTG
O-NF1	-29/-5	CCTGTGGTTTCCTGCCAACAACTCC
O-NF1-mut		CCTGTGGTTTCCT**taa**AACAACTCC
O-Sp1a	+33/+60	GGAGGGGCAGTGGGGCTGGGCCTGGGTG
O-Sp1a-mut		GGAGGGGCAGTGG**tt**CTGGGCCTGGGTG
O-Sp1b	+63/+90	CCCACCAGCCCCGCCCCATCTATCTTTG
O-Sp1b-mut		CCCACCAGCCCCG**aa**CCATCTATCTTTG

**Table 2:** Binding site mutants. Positions of mutations in mutant constructs. Schematic illustration of the *P2X*_1 _core promoter mutation constructs. "." indicates positions same as wild type. "~" indicates where sequence has been omitted for clarity.

	NF-κB	NF-1	Sp1a	Sp1b
Wild type	GGGGAGTCCCC~~~TGGTTTCCTGCCAAC~~~GGGGCTGGG~~~CCCCGCCCC			
mNFkB	..CC.......~~~...............~~~.........~~~.........			
mNF-1	...........~~~.........TAA...~~~.........~~~.........			
mSp1a	...........~~~...............~~~..TT.....~~~.........			
mSp1b	...........~~~...............~~~.........~~~.....AA..			
mut1	..CC.......~~~.........TAA...~~~.........~~~.........			
mut2	...........~~~...............~~~..TT.....~~~.....AA..			
mut3	...........~~~.........TAA...~~~.........~~~.....AA..			
mut4	...........~~~.........TAA...~~~..TT.....~~~.....AA..			

**Table 3:** Oligonucleotides used for ChIP analysis. Primer sequences are 5' to 3'. At the end of oligonucleotide names, f: indicates a sense primer, r: indicates an antisense primer.

**Name**	**Sequence**	**Product size**
P2X_1_f	CGCACGCGTTCTCATTTTGGCAGTGGAGGGGAG	360 bp
P2X_1_r	GGTGATCAGAGCAAAGCTTGGCCCCTGG	
Hsp70f	CCTCCAGTGAATCCCAGAAGACTCT	282 bp
Hsp70r	TGGGACAACGGGAGTCACTCTC	
H4f	GAGAGGGCGGGGACAATTGA	362 bp
H4r	TTGGCGTGCTCGGTGTAGGT	
GAPDHf	TGAAGGTCGGAGTCAACGGATTTGGT	983 bp
GAPDHr	CATGTGGGCCATGAGGTCCACCAC	
